# High-Throughput Digital Image Analysis Reveals Distinct Patterns of Dystrophin Expression in Dystrophinopathy Patients

**DOI:** 10.1093/jnen/nlab088

**Published:** 2021-09-08

**Authors:** Silvia Torelli, Domenic Scaglioni, Valentina Sardone, Matthew J Ellis, Joana Domingos, Adam Jones, Lucy Feng, Darren Chambers, Deborah M Eastwood, France Leturcq, Rabah Ben Yaou, Andoni Urtizberea, Pascal Sabouraud, Christine Barnerias, Tanya Stojkovic, Enzo Ricci, Maud Beuvin, Gisele Bonne, Caroline A Sewry, Tracey Willis, Richa Kulshrestha, Giorgio Tasca, Rahul Phadke, Jennifer E Morgan, Francesco Muntoni

**Affiliations:** 1 From the Dubowitz Neuromuscular Centre, UCL Great Ormond Street Institute of Child Health, London, UK; 2 NIHR Great Ormond Street Hospital Biomedical Research Centre, UCL Great Ormond Street Institute of Child Health & Great Ormond Street Hospital for Children NHS Foundation Trust, London, UK; 3 Department of Neurodegenerative Diseases, UCL Queen Square Institute of Neurology, London, UK; 4 School of Cancer Sciences, University of Southampton, Southampton, UK; 5 Dubowitz Neuromuscular Centre, UCL Queen Square Institute of Neurology & Great Ormond Street Hospital for Children NHS Foundation Trust, London, UK; 6 Department of Orthopaedics, Great Ormond Street Hospital, London, UK; 7 The Royal National Orthopaedic Hospital, Stanmore and University College London, London, UK; 8 APHP, Laboratoire de Génétique et Biologie Moléculaire, HUPC Hôpital Cochin, Paris, France; 9 APHP-Sorbonne Université, Centre de Référence Maladies Neuromusculaires Nord/Est/Ile de France, Institut de Myologie, GH Pitié-Salpêtrière, Paris, France; 10 Sorbonne Université, Inserm, Institut de Myologie, Center de Recherche en Myologie, Paris, France; 11 APHP-Hôpital Marin de Hendaye, Hendaye, France; 12 CHU de Reims—American Memorial Hospital, Reims, France; 13 Department of Pediatric Neurology, Necker Enfants Maladies Hospital, Paris, France; 14 Institute of Neurology, Catholic University, Rome, Italy; 15 Wolfson Centre for Inherited Neuromuscular Diseases and Department of Musculoskeletal Histopathology, RJAH Orthopaedic Hospital, Oswestry, UK; 16 UOC di Neurologia, Fondazione Policlinico Universitario A. Gemelli IRCCS, Rome, Italy

**Keywords:** Becker muscular dystrophy, Duchenne muscular dystrophy, Dystrophin, High–throughput digital analysis, Muscle biopsy, Skeletal muscle

## Abstract

Duchenne muscular dystrophy (DMD) is an incurable disease caused by out-of-frame *DMD* gene deletions while in frame deletions lead to the milder Becker muscular dystrophy (BMD). In the last decade several antisense oligonucleotides drugs have been developed to induce a partially functional internally deleted dystrophin, similar to that produced in BMD, and expected to ameliorate the disease course. The pattern of dystrophin expression and functionality in dystrophinopathy patients is variable due to multiple factors, such as molecular functionality of the dystrophin and its distribution. To benchmark the success of therapeutic intervention, a clear understanding of dystrophin expression patterns in dystrophinopathy patients is vital. Recently, several groups have used innovative techniques to quantify dystrophin in muscle biopsies of children but not in patients with milder BMD. This study reports on dystrophin expression using both Western blotting and an automated, high-throughput, image analysis platform in DMD, BMD, and intermediate DMD/BMD skeletal muscle biopsies. Our results found a significant correlation between Western blot and immunofluorescent quantification indicating consistency between the different methodologies. However, we identified significant inter- and intradisease heterogeneity of patterns of dystrophin expression in patients irrespective of the amount detected on blot, due to variability in both fluorescence intensity and dystrophin sarcolemmal circumference coverage. Our data highlight the heterogeneity of the pattern of dystrophin expression in BMD, which will assist the assessment of dystrophin restoration therapies.

## INTRODUCTION

Duchenne and Becker muscular dystrophy (DMD/BMD) are progressive X-linked neuromuscular disorders that together affect 1 in 3500–5000 newborn males worldwide. They are caused by mutations in the *DMD* gene which lead to absent (DMD) or decreased expression (BMD) of the dystrophin protein and consequently damage and eventually loss of muscle (1). The 2 forms of disease differ in their severity, age of onset, and rate of progression. DMD patients typically live into their 20s. While life expectancy in patients with BMD is closer to that of the general population, a wide variability of severity and outcomes exists. Intermediate clinical phenotypes (IMD) between DMD and BMD are also recognized. Following the implementation of recent standards of care, DMD males are now living into their 30s and in some cases even longer. Similarly, a more proactive approach to prevent respiratory insufficiency and cardiac failure has improved outcomes in BMD.

The *DMD* gene encodes for multiple dystrophin isoforms that are differentially expressed in different organs (brain, eye, smooth, cardiac, and skeletal muscles). However, Dp427m is the predominant isoform expressed in skeletal muscle and its deficiency is responsible for the progressive muscle degeneration in DMD (2, 3). Dystrophin is located in the cytoskeleton just under the sarcolemma of myofibers and forms part of a protein complex, known as the dystrophin-associated protein complex (DAPC). Dystrophin is important not only in connecting the internal contractile apparatus to the extracellular matrix, but also has a crucial signaling role (4).

There is currently no cure for the dystrophinopathies but in the last 10 years several therapeutic approaches aimed at inducing the production of dystrophin protein have been investigated (5).

While it is well recognized that BMD patients with different dystrophin mutations express different level of protein as detected by Western blot (WB), no study has analyzed the dystrophin pattern in muscle biopsies from patients with different clinical severities. This missing information could be important when assessing the biochemical efficacy of a new drug/intervention. This is critical because dystrophin production is now considered an FDA (U.S. Food and Drug Administration) biomarker for conditional approval of DMD drugs (https://www.fda.gov/media/92233/download).

Considerable work has been done in the last few years to develop more sensitive and unbiased techniques to quantify dystrophin determination in muscle. Early studies investigated dystrophin production in DMD and BMD patients using traditional WB (6, 7). In the study by Anthony et al the relatively small population of BMD patients had less than 40% dystrophin expression compared to controls and a correlation between dystrophin levels and disease severity was found (6). However, a subsequent larger study by van den Bergen et al did not find a correlation between dystrophin levels and disease severity in BMD patients, but suggested a threshold effect with levels below 10% indicative of a more severe disease course (7).

Novel related techniques have also been explored, such as ProteinSimple capillary immunoassay (Wes) (3, 8, 9) and mass spectroscopy (MS) (10). While Wes and MS methods have the advantages of measuring very small amounts of protein, being free of gel and blotting hurdles (Wes), and achieving more reliable absolute quantification (MS), they do not give information about dystrophin localization and expression at the sarcolemma, which is important for assessing the molecular functionality of the protein.

Importantly, the immunohistochemical techniques to measure dystrophin production using unbiased quantifiable methods have evolved considerably only in the last few years, and the development of a robust protocol for accurate measurement has been a longstanding goal of the scientific community (11, 12). The first efforts in this field used images of immunostained sections acquired with conventional or confocal microscopy and performed semiquantitative analysis of regions of interest (13–15). Additionally, the analysis in most studies was limited to regions of interest, even if images of entire muscle sections were acquired. This was due to the technical challenges of achieving suitable, artefact free, whole section scanned images (16, 17).

Moreover, recently an immune-mass spectrometry imaging method, based on the detection of a dystrophin antibody conjugated to gadolinium, has been tested but so far only on healthy and some DMD muscle sections with the only data reported expressed as gadolinium concentration (18). We recently published a study in which an image analysis script based on Definiens Developer XD software was developed (19). This method enabled high-throughput, operator-independent assessment of sarcolemmal intensity of dystrophin in intact myofibers in transverse sections. More recently, we modified the script to improve further the acquisition parameters and overall accuracy of dystrophin quantification. In addition, secondary modules were implemented to automatically assess the levels of myofiber regeneration and the expression pattern of proteins of the DAPC in discrete dystrophin-positive or dystrophin-negative sarcolemmal regions (20).

Here, we have used the revised script (20) to investigate the dystrophin expression patterns, and quantity of dystrophin in skeletal muscle biopsies of BMD, IMD, and DMD patients. The aim of this study is to contribute to a better characterization of dystrophin expression patterns in patients with residual dystrophin expression. Since the primary endpoint for many DMD therapeutical approaches is the induction of partially functional internally deleted, BMD-like dystrophins (5), to properly benchmark the success of therapeutic intervention, a better and more precise understanding of dystrophin expression is needed.

## MATERIALS AND METHODS

This study was performed under approval by the NHS National Research Ethics Committee (REC reference number: 13/LO/1894).

In total, 14 biopsies were recruited for analysis from diagnostic archives and patient records reviewed from the following centers: Dubowitz Neuromuscular Centre at the UCL Great Ormond Street Institute of Child Health & Great Ormond Street Hospital for Children, London, UK; Institut de Myologie, G.H. Pitié-Salpêtrière, Paris, France; Laboratoire de Génétique et Biologie Moléculaire, HUPC Hôpital Cochin, Paris, France; Wolfson Centre for Inherited Neuromuscular Diseases, RJAH Orthopaedic Hospital, Oswestry, UK; and Institute of Neurology, Catholic University, Rome, Italy.

### Criteria for Patient Selection

Patients were classified as having mild or severe BMD according to the age at onset, relevant history, and overall motor function throughout the disease course. Mild BMD was defined as having mild proximal muscle weakness but retaining running ability beyond 16 years old. Individuals who either lost running ability before 16 years old or never ran were classified as severe BMD. IMD and DMD patients were classified according to their age at loss of ambulation: DMD before 13 years and IMD between 13 and 16 years old.

### Muscle Biopsies

Diagnostic skeletal muscle biopsies were obtained from BMD/IMD/DMD patients after informed consent (Table 1). Histologically normal muscle biopsy controls (CTRLs) were obtained from the Medical Research Council Centre for Neuromuscular Diseases Biobank (REC reference number: 14/SC/1128).

**TABLE 1.** Summary of Clinical Features

Patient Number and Phenotype	Mutation; IF/OOF	Biopsy Site	Age at Biopsy (y)	Age at Onset (y)	Symptoms at Onset	Motor Functions
1 Mild BMD	del 45–47; IF	Quadriceps	8	5	Waddling gait	Lost ability to run > 16 y; calf and quadriceps atrophy, lower limb proximal weakness (particularly quads), climbs stairs with support.
2 Mild BMD	del45–47; IF	Deltoid	16	8	Waddling gait	lower limb proximal weakness, climbs stairs with difficulties;
3 Mild BMD	del45–49; IF	Quadriceps	6	1–2	Difficulty in climbing stairs/rise from floor; incidental finding (raised CK)	Lost ability to run > 16 y
						Last assessment 21 y: no weakness, mild Tibialis anterior contractures, runs slowly; mild DCM
4 Severe BMD	del45–49; IF	Unknown	3	3	Incidental findings; severely autistic	Lost ability to run <16 y
5 Severe BMD	Point mut exon37-c.5563_5564delCA, p. Gln1855Aspfs*4; OOF	Deltoid	12	6.5	Difficulty in climbing stairs/rising from the floor; delayed walking; cramps	Lost ability to run <16 y, lost ambulation at 21 y
6 IMD	del ex3–7; OOF	Quadriceps	9	unknown	Muscle weakness	Problems running and difficulty getting up off the floor. Unable to hop and difficulty climbing stairs.
7 IMD	del45; OOF	Deltoid	8	3	Calf hypertrophy; tiptoe walk; waddling gait	Ambulant at 17 y but not running, difficulty climbing stairs
8 IMD	Point mut. intron 32 c. IVS33 + 1G>A (c.4674 + 1G>A); OOF	Unknown	18	7	Difficulty in climbing stairs/rise from floor; waddling gait	Lost ambulation at 14 y
9 DMD	exon10-c.1235delT, p. Leu412Tyrfs*13; OOF	Quadriceps	6	<5.6	Weakness	Lost ambulation at 9.9 y
10 DMD	del44; OOF	Quadriceps	4	2	Delayed walking	Lost ambulation at 11 y
11 DMD	del45; OOF	Quadriceps	3	2	Frequent falls	Lost ambulation at 11 y
12 DMD	Point mutation exon20 c.2701G>T, p. Gly901*; OOF	Quadriceps	6	1	Difficulties climbing stairs and rising from the floor	Lost ambulation at 7 y
13 DMD	Del ex 8 -43; OOF	Unknown	7	3	Waddling gait	Lost ambulation at 7.7 y
14 DMD	ex66-c.9748G>T, p. Glu3250*; OOF	Unknown	5	0.5	Delayed walking	Lost ambulation at 6.8 y

Abbreviations: BMD, Becker muscular dystrophy; CK, creatine kinase; DMD, Duchenne muscular dystrophy; IMD, intermediate muscular dystrophy; IF, in-frame; OOF, out-of-frame; DCM, dilated cardiomyopathy; y, years.

### Immunohistochemistry

Muscle biopsies were processed in each center, described above, using standard techniques and either frozen muscle blocks or sections were shipped to the Dubowitz Neuromuscular Centre on dry ice for further analysis. Transverse sections from the muscle blocks were then cut at the Dubowitz Neuromuscular Centre and stored at −80°C before use. Unfixed frozen (5 µm) sections were taken from −80°C and dried at room temperature (RT) for 30 minutes then incubated with a cocktail of primary antibodies: rabbit polyclonal anti-dystrophin ab15277 (1:200, Abcam, Cambridge, UK) raised against the C-terminus and rat monoclonal anti-laminin α-2 (4H8-2, 1:50, Enzo Life Science, Exeter, UK) diluted in phosphate buffered saline (PBS) for 1 hour at RT. After 3 washes in PBS for 3 minutes each, sections were incubated with a cocktail of secondary antibodies (Alexa Fluor-488 goat anti-rabbit IgG and Alexa Fluor-568 goat anti-rat IgG, ThermoFisher Scientific, Waltham, MA) for 30 minutes at RT. After washes in PBS the slides were mounted using Hydromount (National Diagnostics, Nottingham, UK). Two serial sections were immunostained and analyzed for each sample. Whole slide images of entire muscle sections were captured with a ZEISS Axio Scan.Z1 slide scanner (Zeiss, Oberkochen, Germany) and an Orca Flash 4.0 V2 camera. The 16-bit images were acquired resulting in a 0-65536AU fluorescence intensity range. Exposure settings were established in a previous study (20).

### Digital Image Analysis

Images were analyzed by an in-house script developed in Definiens Developer XD (version 2.7.0, Munich, Germany). A detailed review of the image analysis method can be found in our previous publication (20). In brief, laminin-α2 was used as a mask stain to identify the sarcolemma of transverse myofibers. Once the sarcolemmal region had been identified, various parameters of dystrophin expression were then assessed. These included the mean fluorescence intensity of dystrophin staining within the defined sarcolemmal region, the percentage of dystrophin-positive myofibers within each section, and the percentage of sarcolemmal coverage of dystrophin expression for each individual myofiber. Each myofiber was subsequently classified into 1 of 4 categories according to the circumference positivity of dystrophin expression: 0%–25%, 25%–50%, 50%–75%, and 75%–100% coverage. Fibers with 0%–25% positivity were classified as dystrophin negative while fibers with >25% coverage as “dystrophin-positive.” The Mann–Whitney test was used for statistical analysis; significance was set at p = 0.05.

### Western Blot

Muscle samples from either snap frozen tissue or cryostat sections for patients 1, 2, 3, 5, 10, 11, 12, and 13 were solubilized in lysis buffer (urea 4M, Tris 125 mM pH6.8, SDS 4%) containing protease and phosphatase inhibitors (Roche, Merck, Hertfordshire, UK). Protein quantification was performed using the Pierce BCA kit (ThermoFisher Scientific). For each sample 30 ug proteins were loaded on a Nu-PAGE 3%–8% tris-acetate gradient gel (ThermoFisher) and transferred onto nitrocellulose membrane (Amersham Protran, GE Healthcare, Buckinghamshire, UK). Membranes were blocked in 10% nonfat milk (cat no. LP0031, OXOID, ThermoFisher) TRIS buffered saline + 0.1% Tween20 (TBS-T) for 1 hour at RT and then incubated with primary antibodies: rabbit anti-dystrophin (1:200, cat no. ab15277, Abcam) and mouse anti-sarcomeric-α-actinin 2 (1:10 000, cat no. A7811, Sigma, St Louis, MO) overnight at 4°C, in 5% nonfat milk TBS-T. Following washes with TBS-T, membranes were incubated with secondary antibodies, donkey anti-rabbit IRDye 680RD (Li-Cor, Lincoln, NE) and donkey anti-mouse IRDye 800CW (Li-Cor) for 1 hour at RT in TBS-T. After washes with TBS-T, membranes were imaged using the Odyssey infrared imaging system (Li-Cor). WB quantification was performed using Image Studio software (Li-Cor). Results are shown as dystrophin intensity normalized to α-actinin and expressed as percentage of control. Four repeats were run for each patient lysate. The Mann–Whitney test was used for statistical analysis; significance was set at p = 0.05.

## RESULTS

Patients with BMD (n = 5, including 3 mild and 2 severe patients), DMD (n = 6) and IMD (n = 3) along with nonmyopathic, histologically normal controls (n = 2) were included in this study. The average age in each group was 9 **±** 5 years for the BMD, 12 ± 6 years for IMD and 5 ± 1 years for DMD. Biopsies were taken from the quadriceps (n = 7), deltoid (n = 3) and for 5 cases the site of the biopsy was not documented as indicated in Table 1.

All patients in this study had a confirmed genetic diagnosis of dystrophinopathy with different types of mutations including in-frame and out-of-frame single or multiple exon deletions, nonsense mutations or other small mutations. The full list of mutations is summarized in Table 1. The 2 control muscle biopsies (one from the vastus lateralis and the other from the quadriceps) used in this study were from pediatric patients (aged 7 and 8 years) who underwent orthopedic surgery and in whom a primary neuromuscular disease was excluded.

### Dystrophin Intensity in Muscle Fibers

**FIGURE 1.**
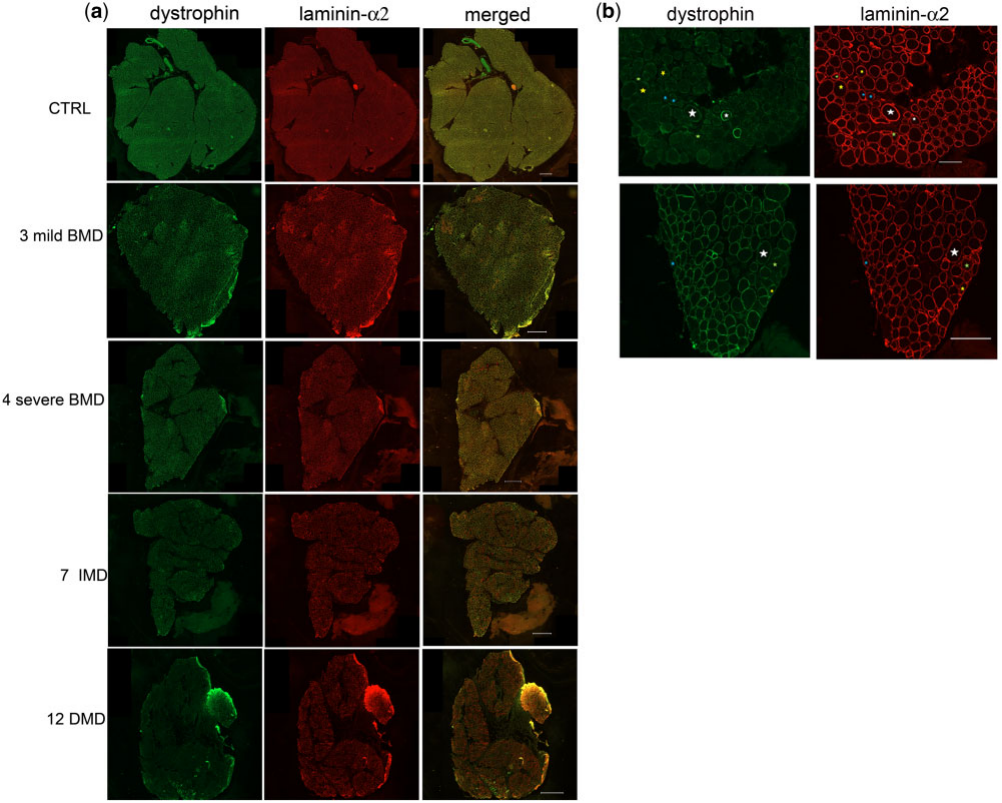
(**A**) Representative images for each phenotype of entire transverse muscle section immunostained with anti-dystrophin (green) and anti-laminin α-2 (red) antibodies. Magnification bar: 500 µm. (**B**) Representative images (upper part, enlarged area from image of patient 11DMD, magnification bar 100 µm; lower part, enlarged area from image of patient 7IMD, magnification bar 500 µm) showing different dystrophin sarcolemmal coverage identified by the script: white star: 75%–100% fiber coverage, yellow star: 50%–74% fiber coverage, blue star: 25%–49% fiber coverage, green star: <25% fiber coverage.

**FIGURE 2.**
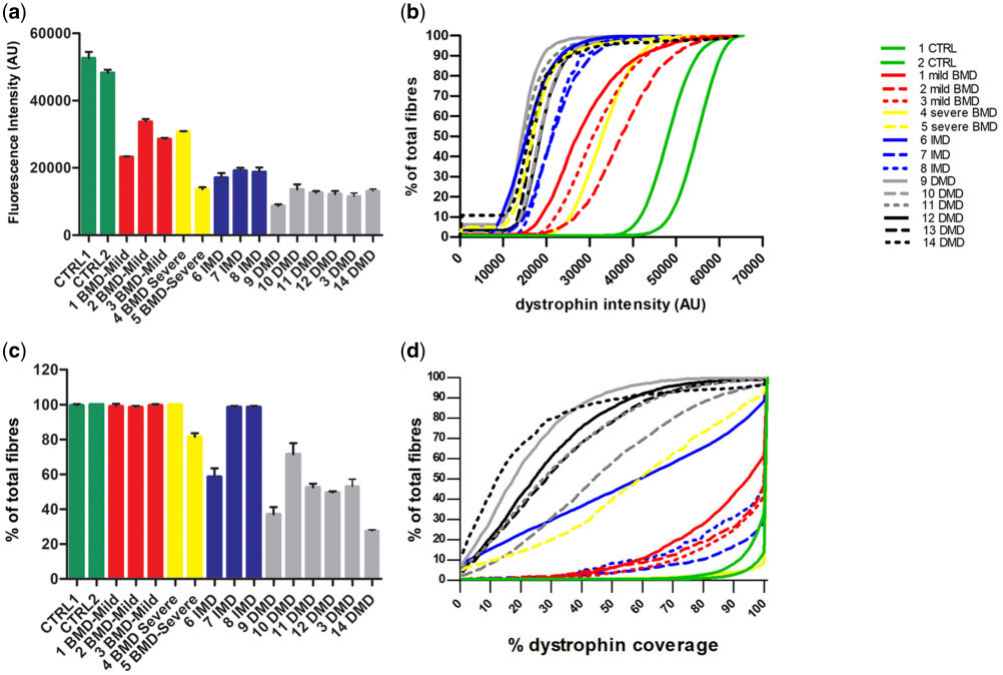
(**A**) Comparative immunostaining analysis of dystrophin expression in patients with BMD, IMD, and DMD. Transverse muscle sections were double labeled with anti-dystrophin and anti-laminin α-2 antibodies. Images of the entire sections were acquired with a ZEISS Axio Scan.Z1 slide scanner and analyzed by a script developed in Definiens. Values represent the dystrophin mean fluorescent intensity (expressed as arbitrary units, AU) ± standard deviation (SD) (error bar) for each patient. (**B**) Cumulative frequency distributions of dystrophin intensity. The lines represent the dystrophin intensity distribution in the fiber population for each patient. (**C**) Percentage of dystrophin-positive fibers in the entire transverse muscle section in individuals with BMD, IMD, and DMD. Values are expressed as percentage of total positive fibers ± SD (error bar). The script classifies as positive a myofiber immunolabeled with dystrophin antibody at the sarcolemma for 25% or more of its circumference. A few patients (1, 2, 3, 4, 7, and 8) showed a percentage of positive fibers similar to controls. The other patients had lower values with patient 14 having the lowest (29.5 + 0.7). (**D**) Cumulative frequency distributions of sarcolemmal circumference coverage of dystrophin. Lines represent the dystrophin coverage distribution in the fiber population for each patient.

Immunofluorescent dystrophin expression was assessed in muscle biopsies from all individuals by applying ab15277 (Abcam), an antibody produced against the C-terminus of human dystrophin (exons 77–79). The entire muscle sections were scanned, their images analyzed, and morphological features and staining profiles of individual muscle fibers recorded according to Scaglioni et al (20). Representative whole section images for each clinical phenotype are shown in Fig. 1A. The average mean dystrophin fluorescence intensity, measured in arbitrary units (AU), and its standard deviation, were recorded for the whole section of each individual. The results are summarized in Fig. 2A and Table 2A.

**TABLE 2.** Dystrophin Results

	Mutation; IF/OOF	(A) Mean Dystrophin Intensity (AU) ± SD	(B) Percent Positive Dystrophin Fibers	WB Percentage of CTRLs
CTRLs		50387 **±** 2778	99.5 **±** 0.6	100
1 Mild BMD	del 45–47; IF	23228 **±** 185	99 **±** 1.4	54
2 Mild BMD	del45–47; IF	33684 **±** 836	98.5 **±** 0.7	54
3 Mild BMD	del45–49; IF	28519 **±** 375	99.5 **±** 0.7	76
4 Severe BMD	del45–49; IF	30798 **±** 86	100 **±** 0	ND
5 Severe BMD	Point mut exon37-c.5563_5564delCA, p. Gln1855Aspfs*4; OOF	13657 **±** 589	81.5 **±** 2.1	17
6 IMD	del ex3-7; OOF	17064 **±** 1367	75.5 **±** 3.5	ND
7 IMD	del45; OOF	19168 **±** 860	98.5 **±** 0.7	ND
8 IMD	Point mut. intron 32 c. IVS33 + 1G>A (c.4674 + 1G>A); OOF	18726 **±** 1393	98.5 **±** 0.7	ND
9 DMD	exon10-c.1235delT, p. Leu412Tyrfs*13; OOF	8694 **±** 498	37 **±** 4.2	ND
10 DMD	del44; OOF	13551 **±** 1568	71.5 **±** 6.4	0
11 DMD	del45; OOF	12572 **±** 552	52.5 **±** 2.1	12
12 DMD	Point mutation exon20 c.2701G>T, p. Gly901*; OOF	12168 **±** 961	49.5 **±** 0.7	0
13 DMD	Del ex 8 -43; OOF	11539 **±** 988	53 **±** 4.2	0
14 DMD	ex66-c.9748G>T, p. Glu3250*; OOF	13161 **±** 529	29.5 **±** 0.7	ND

(A) Dystrophin mean intensity expressed in arbitrary units (AU) ± standard deviation (SD) for each patient (average of 2 experimental replicates). (B) Percent **±** SD of dystrophin-positive fibers for each patient compared to controls. In A and B, the values for the pediatric CTRLs (controls) were the averages of 2 patients without neuromuscular disease. ND, not done; WB, western blot.

There was a significant difference between the mean dystrophin intensity of the CTRLs (mean: 50 387 AU) and both the mild BMDs (mean of 28 477 AU) and the severe BMDs (mean 22 227 AU), (p = 0.0095 and 0.0286, respectively). When comparing dystrophin intensity between different categories of dystrophinopathy patients, we found no difference in mean intensity between mild and severe BMDs. IMDs showed a lower dystrophin mean intensity compared to CTRLs (18 319 AU) and the difference was statistically significant (p = 0.0095). IMDs’ mean dystrophin intensity compared to both mild (28 477 AU) and severe BMD (22 227 AU) was lower; however, the difference between IMDs and BMDs was significant only when compared to the mild BMDs (p = 0.0022) (Table 3A).

**TABLE 3.** Clinical Correlations

Clinical Groups	(A) Mean Dystrophin Intensity (AU) ± SD	p Value	(B) % Dystrophin-Positive Fibers ± SD	p Value
		(Mann–Whitney Test)		(Mann–Whitney Test)
CTRLs vs mild BMDs	50387 **±** 2778 vs 28477 **±** 4695	0.0095	99.5 **±** 0.6 vs 99 **±** 1	NS
CTRLs vs severe BMDs	50387 **±** 2778 vs 22227 **±** 9902	0.0286	99.5 **±** 0.6 vs 90.75 **±** 11	NS
CTRLs vs IMDs	50387 **±** 2778 vs 18319 **±** 1376	0.0095	99.5 **±** 0.6 vs 85 **±** 21	0.0190
CTRLs vs DMDs	50387 **±** 2778 vs 11947 **±** 1800	0.0011	99.5 **±** 0.6 vs 49 **±** 14	0.0011
BMDs vs DMDs	25977 **±** 7740 vs 11947 **±** 1800	<0.0001	96 **±** 8 vs 49 **±** 14	<0.0001
mild BMDs vs severe BMDs	28477 **±** 4695 vs 22227 **±** 9902	NS	99 **±** 1 vs 90.75 **±** 11	NS
BMD all vs IMDs	25977 **±** 7740 vs 18319 **±** 1376	NS	96 **±** 8 vs 85 **±** 21	NS
DMDs vs IMDs	11947 **±** 1800 vs 18319 **±** 1376	0.0001	49 **±** 14 vs 85 **±** 21	0.0017
mild BMDs vs IMDs	28477 **±** 4695 vs 18319 **±** 1376	0.0022	99 **±** 1 vs 85 **±** 21	NS
severe BMDs vs IMDs	22227 **±** 9902 vs 18319 **±** 1376	NS	90.75 **±** 11 vs 85 **±** 21	NS

(A) Comparisons of the dystrophin mean intensity expressed in AU **±** SD; (B) comparisons of the percentage dystrophin-positive fibers **±** SD among the clinical phenotype and their p values. NS, not significant.

In the DMD population, there was a significant difference in the mean intensity between DMDs (mean intensity of 11 947 AU) and CTRLs and both IMDs and BMDs (mild + severe) (p = 0.0011, 0.0001, and <0.0001, respectively, Table 3A).

These intensity results can also be depicted as cumulative frequency distributions (Fig. 2b). In the graph, DMD and BMD samples clearly cluster in different areas with patterns that can be easily distinguished. Interestingly, IMD and DMD individuals have a very similar pattern of dystrophin expression. Among the BMDs, patient 4’s curve (severe BMD, continuous yellow line) is similar to the curve of the milder BMD patient 3 (who shares the same exon 45–49 deletion); their dystrophin mean intensities are 30 798 AU, for patient 4 and 28 477 AU average for the mild BMDs.

### Percentage of Dystrophin-Positive Fibers

Along with intensity, we also assessed the percentage of dystrophin-positive myofibers in each patient’s biopsy sample. A dystrophin-positive fiber was classified as a myofiber containing equal or greater than 25% dystrophin circumference coverage at the sarcolemma. The results for the whole section of each patient are shown in Fig. 2C and Table 2B. Using this parameter, we found that there was a significant difference in the percentage of dystrophin-positive fibers between CTRLs versus DMDs (p = 0.0011), CTRLs versus IMD (p = 0.0190) and BMDs versus DMDs (p < 0.0001) and DMDs versus IMDs (p = 0.0017), but not between BMDs versus IMDs (Table 3B).

### Dystrophin Sarcolemmal Coverage

Myofiber sarcolemmal circumference coverage of dystrophin can also be visualized as a cumulative frequency graph (Fig. 2d); in this graph, the population showing 0%–25% dystrophin coverage, excluded in the graph 2c, is included. The cumulative frequency data clearly highlight the individual expression pattern of each individual, with clustering of patient groups readily apparent.

In addition to cumulative frequency, we also stratified the dystrophin-positive fibers according to the percentage of dystrophin coverage at the sarcolemma recognized by the script into 4 separate groups: 0%–24%; 25%–49%; 50%–74%; 75%–100% (Fig. 1b). While in the previous analysis (Fig. 2c) we only considered positive fibers as those having greater than 25% sarcolemmal dystrophin circumference coverage, in this analysis, any fiber, irrespective of the amount of dystrophin positivity at the sarcolemma, was included. In addition, we assessed the mean average intensity in each of these discrete dystrophin coverage groups.

**FIGURE 3.**
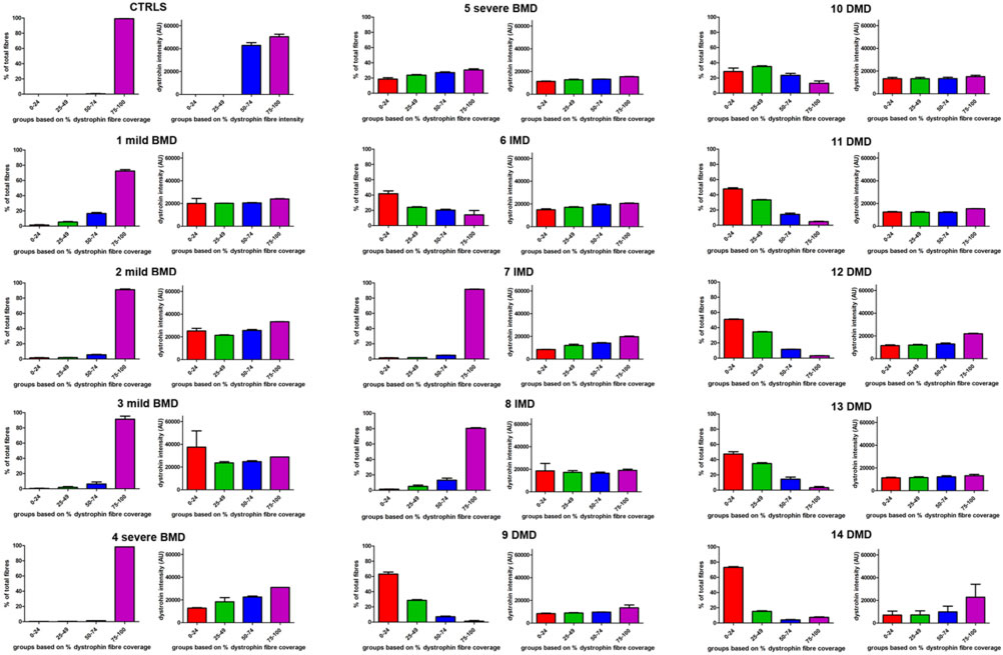
In each section the fiber population was divided in 4 arbitrary groups based on the percentage of dystrophin coverage at the sarcolemma: 0%–24%; 25%–49%; 50%–74%; 75%–100%. The graphs show the percentage of fiber population in each arbitrary group and their mean dystrophin intensity for each patient. For the controls, a representative graph of one of the two controls (CTRL 2) showing the vast majority of the fibers belonging to the 75%–100% group is shown.

We first studied the distribution of dystrophin-positive fibers and the mean dystrophin intensity within each of the 4 categories in 2 controls. As indicated in Fig. 3, 100% of control myofibers were in the 75%–100% sarcolemmal coverage group. The mean dystrophin intensity in the highest group (75%–100%) was 50 418 AU.

Interestingly, all the mild BMDs had similar dystrophin expression patterns, with the majority of fibers having 75%–100% dystrophin coverage (Fig. 3). As expected, the mean dystrophin intensity (28 743 AU) in these fibers was reduced compared to the controls (50 387 AU) despite both having 75%–100% circumference coverage of dystrophin protein. While the mean intensity is approximately half of that in the pediatric control population, the standard deviation is much wider in the BMD patient population, showing that there is greater heterogeneity of dystrophin expression in myofibers of these patients compared to controls.

Analysis of the severe BMDs revealed several different patterns of dystrophin expression. Patient 4 (with a deletion of exons 45–49), and patient 5 (with a frameshift mutation in exon 39), showed different dystrophin coverage patterns (Fig. 3). In patient 4, the pattern was similar to that of mild BMDs with 98% of the fibers in the 75%–100% dystrophin coverage group and a mean intensity of 30798 AU. In patient 5, fibers expressed dystrophin in different percentages at the sarcolemma (19% in group 0%–24%; 24% in group 25%–49%; 27% in group 50%–74%; and 32% in group 75%–100%). This pattern of expression more closely resembles that of the DMD patients (Fig. 3).

Two of the 3 IMD patients, that is, patient 7 (with exon 45 deletion), and patient 8 (with a splice site mutation in intron 33), had a dystrophin sarcolemmal coverage pattern similar to that of the mild BMDs. In contrast, patient 6 (IMD patient with exon 3–7 deletion), showed a pattern similar to a DMD with the highest percentage of fibers (42% ± 5%) in the lowest coverage group and the rest of the fibers spread in the other 3 groups (Fig. 3). However, in all the 3 IMD patients, the mean intensity of dystrophin in the 75%–100% group was very similar (20 519 AU, 19 757 AU, and 19 025 AU, respectively).

In the DMD group, all cases showed a very similar pattern of dystrophin expression (Fig. 3), with most fibers being in the 0%–24% dystrophin coverage group (52% ± 15%), followed by 30% ± 8% fibers in the 25%–49% group, 12% ± 7% in the 50%–74% group and 6% ± 4% in the 75%–100% group. The dystrophin intensity in all 4 groups was also very low compared to controls with an average of 11 252 AU in 0%–24%, 11 484 AU in 25%–49%, 12 561 AU in 50%–74%, and 18 899 AU in 75%–100%.

### Western Blot

**FIGURE 4.**
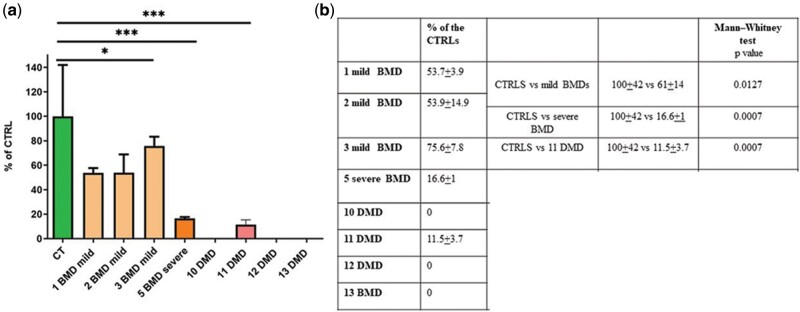
Semiquantitative Western blot (WB) analysis of dystrophin expression in muscle biopsies from BMD and DMD patients. Muscle lysates were run on a 3%–8% tris-acetate gradient gel. Blots were probed with an anti-dystrophin C-terminal and anti-sarcomeric actinin (as a loading control) antibodies. Analysis was performed by Image Studio software (Li-Cor, USA). Results are shown as dystrophin intensity normalized to a-actinin and expressed as percentage of controls. Values represent means of 4 repeats; error bars represent the SD; mild BMD *p = 0.0127; severe BMD ***p = 0.000; DMD ***p = 0.0007.

WB was performed on 8 of the 14 samples for which tissue was available. The results (Fig. 4a), expressed as percentage of dystrophin normalized to controls, show that in only one of the 4 DMD tested samples (DMD 11) it was possible to detect a signal (11.5% ± 3.7%) and with a markedly reduced abundance compared to controls (p = 0.0007). Among the BMD samples, the mild patients showed a relatively high dystrophin expression with an average of 61% that was nevertheless significantly different from the controls (p = 0.0127). The only severe BMD patient (severe BMD number 5) from whom muscle was available for this analysis, showed a 16.6% ± 1% dystrophin expression compared to controls that was also significant (p = 0.0007). Unfortunately, no material from IMD patients was available to perform WB.

**FIGURE 5.**
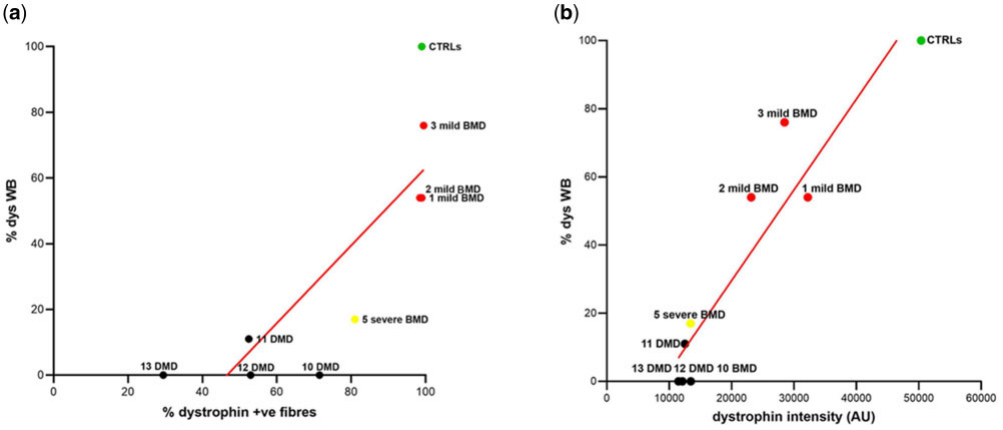
WB correlation with percentage of dystrophin-positive fibers and with dystrophin intensity. The graphs show a good linear correlation between (**A**) WB and mean percentage of dystrophin-positive fiber values (*R* = 0.82) and (**B**) WB and mean dystrophin intensity (*R* = 0.94) in those patients (patients 1, 2, 3, 5, 10, 11, 12, 13) in which it was possible to perform WB.

We then assessed the correlation between WB data and percentage of dystrophin-positive fibers and for WB and dystrophin intensity. We found a good correlation between WB and both the percentage of positive dystrophin fibers (Fig. 5a) and the dystrophin intensity (Fig. 5b) with a R = 0.67 and 0.94, respectively. Clear clustering of clinical phenotypes can also be observed. Interestingly, in both graphs the 4 DMD individuals grouped away from the controls while the 3 mild BMDs are arranged between the controls and the DMD, while the severe BMD is closer to the DMDs than the other mild BMDs.

## DISCUSSION

Several different therapeutic approaches have been described aiming to ameliorate the phenotype of patients affected by DMD. However, detailed information on the patterns of dystrophin expression in patients with BMD and IMD is lacking in the recent studies in which novel technologies to measure protein expression and its quantification are used (17, 21), and most recent studies only used WB (9, 22). In our study, we utilized a high-throughput, operator-independent digital script, previously developed by us, to study a cohort of BMD, IMD, and DMD patients of varying clinical severity. We present an in-depth analysis of the dystrophin expression pattern of these individuals, classifying groups of myofibers based on their percentage circumference coverage of dystrophin and providing detailed assessment of their fluorescence intensity and distribution in comparison to controls and each disease group. Our digital script has the substantial advantage of being based on a multiplex immunofluorescent staining that allows us to collect information on several parameters. It is important to notice that while in a diagnostic setting, immunohistochemical staining is routinely used due to the stability of the chromogenic reporters. But multiplexing using chromogens such as DAB (3,3′-diaminobenzidine) and alkaline-phosphatase has several limitations (i.e. limited option of colors and labor-intensive protocols) (23, 24), making this technique unsuitable for digital imaging.

We also correlate this method of analysis with conventional WB and provide evidence that the 2 methods are well correlated and provide complementary information regarding the quantification of dystrophin. Although WB and quantitative immunocytochemistry are complementary techniques largely used when assessing dystrophin expression especially in clinical trials (17, 21), we believe that our imaging analysis could be used in the routine diagnostic services to provide a more precise and objective evaluation of the different patterns of protein expression compared to the traditional neuropathology reporting methods.

All the DMD muscles studied showed a similar pattern of dystrophin expression, characterized by an average of 49% of positive fibers with a decrease in average intensity of 76% compared to controls. Of these 49% dystrophin-positive fibers, the majority only had percentage of dystrophin coverage at the sarcolemma (25%–50%). IMD individuals showed a high average dystrophin-positive fiber (85% compared to controls), mean dystrophin intensity was markedly decreased compared to the controls and the mild BMDs, while the intensity was similar to the severe BMDs. While variability was observed in the severe BMD and IMD regarding dystrophin coverage, an interesting pattern of expression was detected in 2 IMD patients, one carrying a del45 (7IMD) and another an intron 33 mutation (8IMD). They both showed a high level of dystrophin expression (98% of dystrophin-positive fibers) with a majority (86%) of fibers having 75%–100% coverage, but with significantly reduced intensity (61%) compared to controls. This suggests that having a greater sarcolemmal coverage of dystrophin on the majority of the fibers even if expressed at low intensity may be clinically beneficial, as also suggested by van Wastering et al (25). On the other hand, one of the IMD (6 IMD) in our cohort shows a very similar dystrophin coverage compared to the DMD cluster, yet a comparatively milder phenotype, underlining the complex relationship between amount of dystrophin and its distribution in muscle and phenotypic severity. Indeed, other variables including the functionality of the residual dystrophin protein, secondary to the residual/deleted domains is also likely to play a role, together with other extrinsic factors that are increasingly becoming recognized as modifiers of DMD clinical course (26).

We studied 3 BMD individuals with mild clinical features who showed a very similar pattern of dystrophin expression. Most of the myofibers (85%) in these patients presented with 75%–100% sarcolemmal coverage of dystrophin. As expected, those fibers expressed dystrophin with a lower intensity, 43% less, compared to that of controls. In contrast, the BMD patients with the severe phenotype had a wider range of mean dystrophin intensity values. The severe BMD4 had a similar pattern of expression to the mild BMDs and also shares the same del45–49 mutation as another mild BMD (patient 3). However, the other severe BMD (patient 5), harboring a small mutation in exon 39, showed a dystrophin expression pattern similar to the DMD cohort, with positive fibers present in each coverage group and a similar cumulative frequency distribution curve to DMD and IMD patients. This indicates that the total amount of dystrophin within the muscle is not the only predictive factor of clinical severity and that the distribution of the protein is also important, as these patients produce different BMD-like “dystrophins” in muscle.

Previous studies reported a milder disease course being associated with either higher dystrophin levels, (11, 27), or with a threshold effect (7, 28) while other studies correlated disease severity with dystrophin mutation (29). A more recent study described dystrophin levels as low as <0.5%, associated with a milder phenotype (21). However, no information about localization and distribution of the dystrophin was shown. It is clear that there is a complex relationship between “amount” of dystrophin, its distribution in the muscle, and the “quality” (its molecular functionality via a correct DAPC interaction) of the resulting protein, in relation to the different mutations. Therefore, when attempting to correlate expression patterns with clinical severity, the quantification of both factors will be pivotal for future studies and for benchmarking the efficacy of therapeutic interventions.

The main limitation of this study is the relatively small number of patients analyzed for each phenotype. This was related to the relative rarity of these conditions, to the increasing reliance on genetic testing to arrive at a final diagnosis without performing a muscle biopsy, and the need to have sufficient skeletal muscle, well-preserved, and with the ethical consent available to run these analyses.

While the results in the mild BMD and DMD group were consistent, there is a large spectrum of clinical variability in these conditions, which was reflected in the variability observed in the relatively small number of patients of the severe BMD spectrum and in the IMD group, and it would have been helpful to have additional samples available. Moreover, the average age in each group is not similar; however, there is so far no information in the literature about progressive changes with age in the levels of dystrophin in patients, or in normal muscle, but this cannot be completely excluded.

It should also be noted that biopsies were not all taken from the same muscle group. It has previously been shown that different muscle groups present with varying levels of dystrophin, even within the same patient (19) and therefore this may account for some of the observed variability. Additionally, while several commercial antibodies against different regions/epitopes of dystrophin protein are available, in our study we used only one antibody, a polyclonal raised against the C-terminus of the protein. In the future, studies involving a higher number of individuals, particularly within the severe BMD and IMD spectrum and antibodies against different regions of the dystrophin protein, and ideally involving biopsies samples from the same site, will help in validating these preliminary results and provide a more complete picture of the muscle fiber populations relative to dystrophin composition.

Nevertheless, a clear finding in our study is that the difference between the mild BMD, and the more severe DMD, is related to several variables. These include the level of dystrophin expression, sarcolemmal dystrophin coverage and distribution, and the intensity of the sarcolemmal expression. The milder BMD all clustered together and separate from the IMD and severe BMD both for intensity and for number of positive fibers. Having only one of these parameters in a high value range was not sufficient to provide a significant amelioration of the phenotype, at least in the patient population studied. All these factors contribute to the definition of the muscle pathology observed, demonstrating a higher degree of heterogeneity than can be inferred by a single measurement. Improved understanding of the heterogeneity of dystrophin expression in BMD, IMD, and DMD patients will be vital to investigators who use muscle pathology to benchmark outcomes in future DMD clinic trials.

## Notes

Silvia Torelli and Domenic Scaglioni are co-first authors and contributed equally to this work.

This work was supported by the National Institute for Health Research Biomedical Research Centre at Great Ormond Street Hospital for Children NHS Foundation Trust and University College London and the MRC Centre for Neuromuscular Diseases Biobank.
